# Supplementary material for: Development of an IntelliCage-based cognitive bias test for mice
Source: Open Res Eur. 2023 Aug 8;2:128. Originally published 2022 Nov 25. [Version 2] doi: 10.12688/openreseurope.15294.2 (PMC10548109; doi:10.12688/openreseurope.15294.2)
Supplement: Peer review history

# Reviewer report: Reviewer response for version 2

**DOI:** 10.21956/openreseurope.17783.r34338

**Published:** 2023-10-03

**Authors:** Ewelina Knapska

**Affiliations:** 1 Laboratory of Emotions' Neurobiology, Center of Excellence for Neural Plasticity and Brain Disorders BRAINCITY, Nencki Institute of Experimental Biology, Warsaw, Poland

The authors successfully addressed all my concerns.

Is the rationale for developing the new method (or application) clearly explained?

Yes

Is the description of the method technically sound?

Yes

Are the conclusions about the method and its performance adequately supported by the findings presented in the article?

Partly

If any results are presented, are all the source data underlying the results available to ensure full reproducibility?

Yes

Are sufficient details provided to allow replication of the method development and its use by others?

Yes

Reviewer Expertise:

Behavioral Neuroscience, working with mice and rats. I have been working with the IntelliCage system since 2004.

I confirm that I have read this submission and believe that I have an appropriate level of expertise to confirm that it is of an acceptable scientific standard.

# Reviewer report: Reviewer response for version 2

**DOI:** 10.21956/openreseurope.17783.r34337

**Published:** 2023-08-15

**Authors:** David P. Wolfer

**Affiliations:** 1 Institute of Anatomy, Division Functional Neuroanatomy, University of Zurich, Zurich, Switzerland; 2 Institute of Human Movement Sciences and Sport, D-HEST, ETH Zurich, Zurich, Switzerland

The authors have addressed all my concerns.

Is the rationale for developing the new method (or application) clearly explained?

Yes

Is the description of the method technically sound?

Yes

Are the conclusions about the method and its performance adequately supported by the findings presented in the article?

Partly

If any results are presented, are all the source data underlying the results available to ensure full reproducibility?

Yes

Are sufficient details provided to allow replication of the method development and its use by others?

Yes

Reviewer Expertise:

NA

I confirm that I have read this submission and believe that I have an appropriate level of expertise to confirm that it is of an acceptable scientific standard.

# Reviewer report: Reviewer response for version 1

**DOI:** 10.21956/openreseurope.16536.r30671

**Published:** 2023-02-14

**Authors:** Ewelina Knapska

**Affiliations:** 1 Laboratory of Emotions' Neurobiology, Center of Excellence for Neural Plasticity and Brain Disorders BRAINCITY, Nencki Institute of Experimental Biology, Warsaw, Poland

The manuscript by Pia Kahnau *et al.* describes an attempt to develop a method of measuring cognitive bias in mice using the IntelliCage. The cognitive bias test measures how emotional states affect cognitive processes. The test uses classical or operant conditioning to measure responses to ambiguous cues. It can be used to measure animals’ welfare state, as those in better welfare make more optimistic judgments. The cognitive bias test is well-described in many species, including mice. The novelty of the studies described in the manuscript is using the IntelliCage, an automated cage for assessing mice behavior and learning, to develop a new protocol for measuring cognitive bias. Developing a reliable protocol would be useful as it would be a valuable tool to assess the welfare of the laboratory mice and study mechanisms of cognitive bias. The authors present three experiments through which they came closer to designing optimal test conditions. The methods are well described, and all the necessary experimental details are provided. However, the aim of that study, i.e., the development of a reliable cognitive bias test for mice in the IntelliCage, has not been achieved. Thus, additional experimental work is needed.

 Major comments:

1. The mice were only tested for cognitive bias in the third experiment, in which a shift toward more optimistic decisions was observed after a supposedly stressful procedure. This effect was maintained even when the animals were not stressed, i.e., their responses did not return to the baseline. This result should be carefully considered. One of the possible scenarios the authors did not discuss is that the procedure they used to stress the mice was actually not aversive. Removing some elements of the environment which was already enriched (the mice had access to a large territory, had to learn a new behavior, etc.) and adding very mild restrain changed the behavior of mice in the direction opposite to the predicted one. It could be the effect of positive cognitive bias. Still, the change conditions could also affect learning as the response curve did not return to baseline. I recommend testing the protocol using conditions already known to induce cognitive bias.
2. To show the reliability of the cognitive bias protocol, it should be tested on more than one cohort of mice.
3. As during the training and testing only one mouse could enter the IntelliCage at the same time (when another animal did not already occupy it), it would be good to show, for each animal, how often it entered the training/testing chamber. Such data may explain the relatively high drop-out rate of animals that were unable to learn (if some mice waited for another trial too long, it might have affected memory consolidation).
4. The test has been developed for females only. The authors should discuss how to develop such a test for male mice.

Minor comments:

1. It would be helpful for the reader to get information that mice are individually recognized in the IntelliCage system thanks to the transponders they carry early in the text (e.g., in the Introduction).
2. It needs to be clarified whether, during habituation to nose-poking, all mice had access to the IntelliCage or whether they went there one by one as during the following stages.

Is the rationale for developing the new method (or application) clearly explained?

Yes

Is the description of the method technically sound?

Yes

Are the conclusions about the method and its performance adequately supported by the findings presented in the article?

Partly

If any results are presented, are all the source data underlying the results available to ensure full reproducibility?

Yes

Are sufficient details provided to allow replication of the method development and its use by others?

Yes

Reviewer Expertise:

Behavioral Neuroscience, working with mice and rats. I have been working with the IntelliCage system since 2004.

I confirm that I have read this submission and believe that I have an appropriate level of expertise to confirm that it is of an acceptable scientific standard, however I have significant reservations, as outlined above.

# Response

**Published:** 2023-08-01

**Authors:** Pia Kahnau

**Affiliations:** German Federal Institut for Risk Assessment, Germany

First of all, we would like to thank you for taking the time to review our manuscript in the open review process. The summary is concise and the clearly formulated comments will help to improve the manuscript.

Major comment 1: It is true that the data suggest a more optimistic emotional state due to the supposedly more negative experiences (removal of enrichment and repeated confinement). In rats and also in mice, housing conditions have been shown to influence the cognitive biases of animals. However, in our study, the group of mice used was their own control. Based on the results, one has to ask whether a group of mice can be the control at the same time, whether it is even possible to measure a before-after effect with our experimental design (see discussion on page 28). The extent to which the removal of the enrichment actually has a negative effect on the mice has indeed not been conclusively clarified. However, restraining is a paradigm regularly used to inflict negative experience and we assume that it is not too bold to claim that the restrain test was a negative experience for the mice, at least for the duration of the restraining. We observed that after the first trial on the first day, the mice did not enter the restrain tube voluntarily and showed clear avoidance behavior in the form of reduced interaction with the experimenter’s hand. Unfortunately, we can only report on this behavior anecdotally. However, it could be that the end of the restraint test had a positive effect on the cognitive bias, along the lines of "now everything is fine again", and may even have overridden the influence of the removal of the enrichment. We have added this consideration in the discussion (page 27). It is also possible that the mice learned that nosepoking in response to the ambiguous tones had no consequences. This point was elaborated in the discussion (page 28).

Major comment 2: We agree. Therefore, another study is planned in which two groups (a treatment and a control group) will be tested simultaneously. Nevertheless, in the presented study we were already able to measure the cognitive bias of the mice in an automated and home cage based set-up, although, it was not possible to clearly manipulate the cognitive bias. Nevertheless, we believe it is important to describe the steps involved in measuring the cognitive bias, as this information could potentially be helpful in the development of more home cage-based experiments. We consider this paper as a methods paper rather than a results paper.

Major comment 3: Thank you for this comment. It is theoretically possible that some mice were not able to enter the IntelliCage or were able to enter less frequently and were therefore presented with the tones less often during the conditioning phase. We noticed that all mice were able to enter the IntelliCage several times per night. Once a mouse was in the IntelliCage, it had the ability to repeatedly enter the corner of the IntelliCage and may have been able to compensate for fewer IntelliCage entries. We have added two figures to the supplements showing that the mice were able to enter both the IntelliCage and the IntelliCage corner several times. We assume that the mice that had not learned to discriminate between the tones had an aversion to the possible punishment and not that they had not learned because they could enter the IntelliCage less often (discussion page 18).

Major comment 4: Thank you for mentioning this, it is true that we have only used females so far. The results may not be transferable to males in a one-to-one manner, so it is useful and necessary to test both the set-up and the experiment with males. In the methods section (page 6), we have explained in more detail the need to pay attention to behavior in experiments with males. On the one hand, that individual males do not occupy the IntelliCage and prevent other mice from entering, and on the other hand, agonistic behavior needs to be monitored in order to intervene as quickly as possible in case of injury.  

Minor comment 1: We agree that the information about the individual recognizability of the IntelliCage system through RFID antennas and transponders should already be mentioned in the introduction and helps the reader to understand the functionality of the system. Therefore, we have included this information in the introduction (page 4).

Minor comment 2: That is an important hint, thank you very much. Especially the habituation of the set-up is important and should be described in the manuscript. That is why we have added the information in the methods section (page 8).

# Reviewer report: Reviewer response for version 1

**DOI:** 10.21956/openreseurope.16536.r30672

**Published:** 2023-02-09

**Authors:** David P. Wolfer

**Affiliations:** 1 Institute of Anatomy, Division Functional Neuroanatomy, University of Zurich, Zurich, Switzerland; 2 Institute of Human Movement Sciences and Sport, D-HEST, ETH Zurich, Zurich, Switzerland

Pia Kahnau and co-authors describe the development and validation of a test protocol which allows to assess emotional state in mice using the IntelliCage system. The concept of the test is that in a training stage the mice first learn to discriminate two acoustic stimuli and to associate one of them with a reward (delivery of water) and the other with a punishment (delivery of an air puff). In the subsequent test stage, the mice are confronted with ambiguous stimuli and their bias toward interpreting them as predicting reward or punishment is taken as a readout of their emotional state. This concept of testing emotional state by measuring cognitive bias is well established. New is the presentation of a protocol that works in the automated IntelliCage system which allows to test the mice with minimal handling by humans and without social deprivation. In the first two steps of development, the authors established the training stage of the protocol in which mice reliably and measurably learned to discriminate two acoustic stimuli. In the third step, the test stage of the protocol was developed and tested by confronting mice with ambiguous stimuli after they had been stressed by repeated restraint and sensory deprivation.

 The development of this new protocol is well described and documented. The presented data demonstrate that it has the potential to become a new test of cognitive bias which is as animal-friendly as possible, but a final convincing proof that it works reliably and is indeed suitable for this purpose requires additional experimental work.

**Major points:**

1. My principal concern with this paper is that the conclusion that using the presented protocol the authors “were able to measure and manipulate the cognitive bias” is overstated. As the authors correctly note, further research is needed, but not only to “for a better understanding of the mice’s cognitive bias” but to establish convincingly that that the protocol is indeed suitable for the intended purpose. As already pointed out in the Discussion, there are two major problems with the data. First, there was uncertainty what direction of bias to expect after the stress manipulation used in the experiment. Second, it could not be demonstrated that the mice returned to baseline after recovery. Thus, the possibility remains that the mice learned during establishment of the first baseline that the new stimuli do not predict punishment and therefore showed a positive bias in subsequent tests. So, the observed cognitive bias may reflect learning rather than emotional state. I would suggest to re-test the protocol while (i) using a well-established way to change emotional state such as protocols used in depression models and (ii) using a cross-sectional design in which an experimental and a control group can be directly compared while they are still naïve to the ambiguous stimuli.
2. Only female mice were tested. While testing male subjects is certainly beyond the scope of the present study, it needs to be acknowledged that follow up studies will be needed to demonstrate that the method works independently of sex.

**Minor points:**

1. There was at least one previous attempt to measure cognitive bias in the IntelliCage system, published in Alboni *et al.* (2017 ^1^). I would appreciate it if the authors could critically comment on these attempts in the Introduction.
2. I could not find a formal power analysis of the experimental designs used in this work, but I would assume that with the given numbers of subjects and drop-outs it would be difficult to statistically demonstrate absence of effect. So, negative test outcomes essentially remain inconclusive. Therefore, the authors should tone down their interpretation of negative test outcomes and state that there was no evidence for an effect or that time points/stimuli were indistinguishable instead of claiming that there was no effect.
3. In several instances, the authors merely state that there was a significant effect. They should also give a brief description of what the effect actually was, so that its meaning can be appreciated by the reader. Especially with interaction effects this is not always self-evident.
4. Typos: p20 “It is discussed wheather”, p21 “the period of negative conditions was significantly lower” shorter?
5. I assume that the specific IntelliCage protocols would be made available upon request.

Is the rationale for developing the new method (or application) clearly explained?

Yes

Is the description of the method technically sound?

Yes

Are the conclusions about the method and its performance adequately supported by the findings presented in the article?

Partly

If any results are presented, are all the source data underlying the results available to ensure full reproducibility?

Yes

Are sufficient details provided to allow replication of the method development and its use by others?

Yes

Reviewer Expertise:

Behavioral Neuroscience, mostly using mice as disease models. Have been working with the IntelliCage system for almost 2 decades now.

I confirm that I have read this submission and believe that I have an appropriate level of expertise to confirm that it is of an acceptable scientific standard, however I have significant reservations, as outlined above.

## References

[1] : Fluoxetine effects on molecular, cellular and behavioral endophenotypes of depression are driven by the living environment. *Mol Psychiatry* .2017;22(4) : 10.1038/mp.2015.142 552-561 10.1038/mp.2015.142 26645631PMC5378807

# Response

**Published:** 2023-08-01

**Authors:** Pia Kahnau

**Affiliations:** German Federal Institut for Risk Assessment, Germany

Thank you for the clear summary and for agreeing to review our manuscript in the open peer review process. Your comments address important aspects of the limitations of our work and the editing will help to improve our manuscript. We understand the objection that our claim that we "were able to measure and manipulate the cognitive bias" is overstated and we adjusted our interpretation of the data accordingly.

Major point 1: Regarding the "uncertainty what direction of bias to expect":  In the discussion of developmental step 3 we state that we expect a negative cognitive bias, but we agree that this expectation can already be mentioned earlier. Therefore, we added the sentence "In accordance with studies in rats, we expect the cognitive bias of the mice to be negatively affected by removal" on page 20. Regarding the concern that "it could not be demonstrated that the mice returned to baseline after recovery": It is true, that the mice in the cognitive bias test 1 do not return to the baseline level. This could indeed be an indication of learning. It appears that the mice have learned that they will not be rewarded or punished for the ambiguous tone frequencies. The nosepoke behavior also appears to be higher in the second cognitive bias measurement compared to baseline 1 in the cognitive bias test 1. The mice seem to be nosepoke more overall over time. Whether this is due to a more optimistic emotional state, or whether the mice have learned that ambiguous tones will not be punished, cannot be determined from the data presented here. Rather, it seems that a before and after comparison is not possible in this set-up. The conclusion that one mouse group seems to be unsuitable as its own control was added to the discussion of developmental step 3 (page 28). In a follow-up study, two groups with different experiences will be tested simultaneously and the cognitive bias will be compared.   However, the sigmoidal curve is maintained in each measurement and the mice nosepoke more to the near positive tones than to the mid tones. This suggests that the mice are evaluating whether or not to make nosepokes. If the mice had learned that they would not be punished on the ambiguous tones, I would expect a much higher rate of nosepokes (close to 100%) in response to the ambiguous frequencies, based on experience from other IntelliCage experiments (Kahnau et al 2021). Nevertheless, learning cannot be completely ruled out here, so the claim that we were able to manipulate the cognitive bias was significantly weakened. *Kahnau P, Guenther A, Boon MN, et al.: Lifetime Observation of Cognition and Physiological Parameters in Male Mice. Front Behav Neurosci. 2021;15:709775. 34539359 10.3389/fnbeh.2021.709775 8442583*

Major point 2: We agree that it is necessary to test the set-up and method additionally with males. Therefore, we have added to the methods section on page 6 that male mice should also be tested in this set-up to measure their Cognitive Bias. In addition, we pointed out possible experimental difficulties in tests with male mice in such a set-up.  

Minor point 1: Thank you for pointing out this paper, which we have included in our introduction (page 3) and discussion (page 26, 27). It is true that in Alboni et al. (2017) (among other results) the result of an IntelliCage-based test of cognitive bias are shown. For this test, mice were conditioned to light (blue and yellow). The stimuli were presented every time a corner was visited, as in our study, which had the advantage that the mice could initiate each trial themselves and thus experienced more self-determination, leading to an improvement in the experimental conditions. The focus of this paper was certainly not to describe the development of the method. Therefore, some parameters that would have been helpful for the evaluation of the study are unfortunately missing. For example, neither the paper nor the supplements provide more detailed information on the number of animals that could not be successfully conditioned. Unfortunately, it is also not clear how many IntelliCages were used in total and how many animals were kept as a social group in an IntelliCage. However, the density of a cage could have an effect on learning, as mice can pull, push or shove each other out of corners. A disadvantage of the cognitive bias test shown in this study is that only one ambiguous stimulus is presented, probably due to the fact that the IntelliCage has only three different lights (blue, yellow and red). Because only one ambiguous stimulus is presented, it is not possible to plot the data on a sigmoid curve. In such a plot of the data, it is not possible to see how well the mice discriminate between the conditioned stimuli and how they interpret the ambiguous stimulus. According to Lagisz and colleagues, even a single ambiguous stimulus seems to reduce the sensitivity of the test (Lagisz et al. 2020). Unfortunately, it is also not stated from how many sessions the cognitive bias test results were obtained. Nevertheless, a difference in emotional state was found between two groups of mice. An enriched environment combined with fluoxetine treatment seems to have a positive effect on the emotional state of depressed mice compared to mice that lived in the same environment but were not treated with fluoxetine. We have not yet been able to clearly demonstrate a manipulation of cognitive bias with our method. *Lagisz M, Zidar J, Nakagawa S, et al.: Optimism, pessimism and judgement bias in animals: A systematic review and meta-analysis. Neurosci Biobehav Rev. 2020;118:3–17. 32682742 10.1016/j.neubiorev.2020.07.012*

Minor point 2: As this was an exploratory study, it was not possible to estimate the effect size and power in advance. Especially at the beginning, it was not clear how the mice would behave in this experimental design. We decided on 12 animals per group (and developmental stage) because it is possible to keep 12 mice in a type IV cage and still provide different enrichments. RFID technology also allows individual data to be obtained. We agree that the number of animals used might seem small and that the statements on negative results regarding the results of the cognitive bias tests should be toned down. We did so and we have added some paragraphs under “Animals and housing conditions” (page 5), “discussion” (page 28) elaborating on the group size.

Minor point 3: We thank you for pointing this out. We have described the results more clearly in the results section.

Minor point 4: Thank you for these hints. We have corrected the typos.

Minor point 5: Of course, the IntelliCage protocols will be made available if there is interest.
